# Elucidating the Temporal Patterns of Gene Expression in the Inferred Regulatory Interactions of *GmCOL1b* in *Glycine max*

**DOI:** 10.17912/micropub.biology.000924

**Published:** 2023-11-21

**Authors:** Michelle Alcantara, Hira Iftikhar, Dianna Dzheyranyan, Kimberly Kagan, Pedram Abbasi, Alejandra Alamilla, Nicole Ayala, Trixy Baca, Vanessa Benoit, Natalia Clausen, Caroline Coto, Celia Guerrero, Erik Hernandez Catalan, Sierra Hurtado, Angela Lopez, Jacqueline Lopez, Nicholas Majarian, Noah Mesfin, Avetis Mishegyan, Goharik Mkrtchyan, Amy Ordonez, Arthur Pachanyan, Tanya Pelayo, Alondra Rosas, Kylee Rowsey, Elina Sharma, Sanjiti Sharma, Shauna Van Grinsven, Yoshie Hanzawa

**Affiliations:** 1 Department of Biology, California State University Northridge; 2 Department of Biology, BIOL 481L Plant Physiology, California State University Northridge

## Abstract

The *CONSTANS* ( *CO* ) gene in *Arabidopsis thaliana* has a central role in photoperiodic regulation of flowering. However, the roles of *CO* genes in mediating flowering in soybeans ( *Glycine max* ) remain uncertain. We previously inferred regulatory interactions of a soybean *CO* homolog, *GmCOL1b* , using in-house RNA-seq data and the network inference algorithm package CausNet. Here, we identify potential *GmCOL1b* downstream genes and experimentally verify them by expressing *GmCOL1b* in soybean protoplast cells. Temporal expression patterns of these genes indicate the regulatory effects of *GmCOL1b* on the expression of the circadian clock genes *GmLCL1* and *GmLCL4* and the flowering regulator *GmTEM1a* .

**Figure 1. Expression of GFP-GmCOL1b and inferred downstream genes in GFP-GmCOL1b transfected protoplasts.**
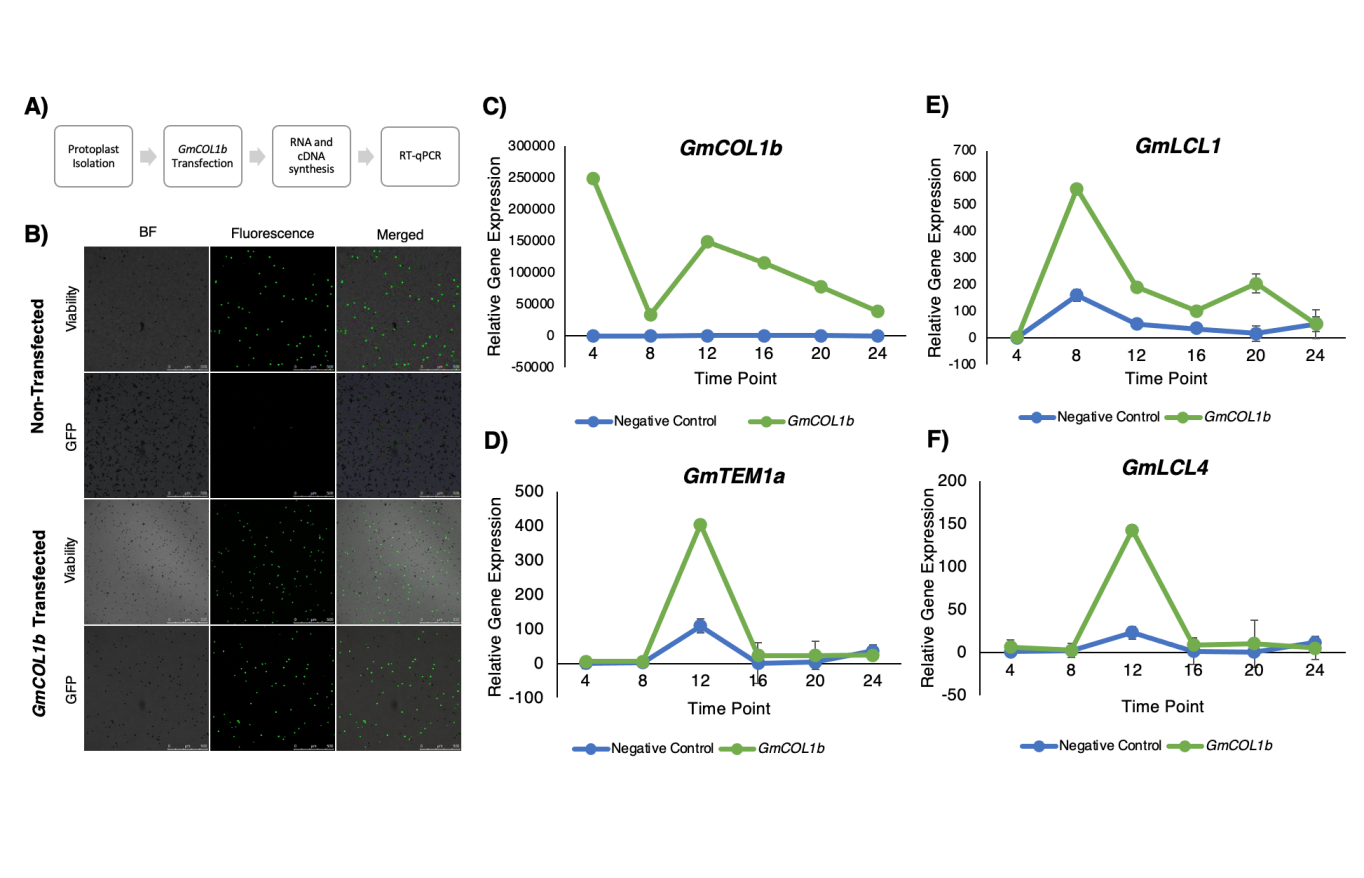
**A)** Flowchart of experimental steps used in this study. **B)** Representative images of non-transfected and *GFP-GmCOL1b* transfected protoplasts in bright field (BF) and fluorescent light at a wavelength of 523nm (514-527nm range) showing cell viability and GFP expression. To determine cell viability, protoplasts were stained with FDA at the time of harvest. **(C-F)** Relative expression of *GmCOL1b* (C) and inferred *GmCOL1b* target genes: *GmTEM1a* (D), *GmLCL1* (E), and *GmLCL4* (F) in non-transfected negative control protoplasts (blue) and *GFP-GmCOL1b* transfected protoplasts (green) at Zeitgeber time points ZT4-ZT24 by RT-qPCR in two biological samples with three technical replications. Normalized expression levels were calculated as 2 ^-∆Ct^ against the housekeeping gene *GmPBB2* as described previously (Livak & Schmittgen, 2001; Wu et al., 2014). Relative gene expression levels were calculated for each graph using the ZT4 expression level in the Negative Control cells as 1. Error bars indicate standard error with 2 biological replicates.

## Description

Soybean ( *Glycine max* ) is a vital legume with multipurpose functionality as human food, animal feed, and biodiesel (Liu et al., 2020) . Soybeans synchronize the timing of reproductive transition (flowering) to a seasonal change in daylength (photoperiod), from long day (LD) to short day (SD) during the summer solstice (Cao et al., 2017; Lin et al., 2021) . Therefore, photoperiodism has a vast influence on flowering control in the SD-plant soybean. However, the molecular basis of the photoperiodic flowering response is poorly understood in soybean.

In the long-day flowering plant *Arabidopsis thaliana* (Arabidopsis), photoperiod-regulated flowering is mediated through the B-box transcription factor CONSTANS (CO) where endogenous rhythms of the circadian clock and external seasonal light/dark lengths coincide (Takagi et al., 2023; Turck et al., 2008) . Among the large number of genes participating in the transcriptional and post-transcriptional regulation of CO, the *CO* mRNA transcription is in part controlled by a component of the circadian clock, GIGANTEA (GI) (Sawa et al., 2007) , as well as by FLAVIN-BINDING KELCH REPEAT, F-BOX 1 (FKF1) and CYCLING DOF FACTOR (CDF) proteins that are controlled by the circadian clock (Imaizumi et al., 2005; Sawa et al., 2007; Song et al., 2012) . Under blue light in LD conditions, GI and FKF1 proteins form a complex in the late afternoon and degrade CDF1 proteins, a family of Dof-type zinc finger transcription factors that repress *CO* transcription (Fornara et al., 2009; Imaizumi et al., 2005) , resulting in the activation of *CO* transcription towards the end of the day in LD. In addition, PSEUDO RESPONSE REGULATOR (PRR) proteins, components of the circadian clock’s central oscillator, are known to stabilize CO proteins at different times of the day (Hayama et al., 2017) . PRR9 is suggested to contribute to the morning peak of CO accumulation, whereas PRR5, PRR7, and TIMING OF CAB2 EXPRESSION 1 (TOC1) stabilize CO in late afternoon in LD conditions. The CO peak exposed to light at the end of the day promotes flowering through the activation of the floral integrator gene *FT* (Song et al., 2012; Valverde et al., 2004) . At night, EARLY FLOWERING 3 (ELF3), a component of the evening complex of the circadian clock, promotes degradation of CO proteins (Song et al., 2018) likely by making a complex with the E3 ubiquitin ligase CONSTITUTIVE PHOTOMORPHOGENESIS 1 (COP1) (Yu et al., 2008) . In a sharp contrast to the extensively studied, elaborate regulatory mechanisms of CO at the transcriptional and post-transcriptional levels, CO’s regulatory roles in the control of the flowering gene network other than its well-known target, *FT* , are largely unclear.

In the short-day flowering plant soybean, the CO-FT module in photoperiodic flowering regulation may be conserved, but with certain modifications (Cao et al., 2015; Wu et al., 2019) . The *CO* homologs *CONSTANS-LIKE 1a* ( *GmCOL1a* ) and the *CONSTANS-LIKE 1b* ( *GmCOL1b* ) appear to act as flowering repressors under LD conditions rather than inducers, by downregulating the *FT* homologs *GmFT2a* and *GmFT5a* that activate flowering under SD conditions, resembling the action of the CO-FT module in rice, another short-day flowering crop (Komiya et al., 2008; Yano et al., 2000) . However, further regulatory roles of *GmCOL1a* / *GmCOL1b* are yet to be discovered.

Similar to Arabidopsis, recent studies have reported that soybean’s circadian clock influences photoperiodic flowering by modulating the *E1* gene, a legume specific flowering repressor (Xia et al., 2012) in twofold. Firstly, the soybean LHY homolog LHY1a directly binds to the promoter of *E1* and inhibits *E1* expression (Lu et al., 2020) . Secondly, components of the evening complex, GmELF3 and GmLUX homologs, directly inhibit *E1* expression (Bu et al., 2021; Lu et al., 2017) . Regulatory interactions of *GmCOL* genes and the circadian clock genes, however, are unknown.

To clarify the regulatory roles of the *CO* gene family in photoperiodic regulation of flowering in soybean, we sought for genes that were controlled by *GmCOL1b* using a network inference approach followed by experimental verifications. The purpose of this study is to gain a clearer picture on the function of *GmCOL1* genes in the flowering gene network that controls soybean’s SD flowering habit by identifying and characterizing genes that may be regulated by *GmCOL1b.*

We inferred regulatory interactions among soybean’s circadian clock and flowering genes using in-house time series RNA-seq data and the network inference package CausNet (Alcantara et al., 2022; Wu et al., 2019) . Inferred regulatory interactions were visualized on Cytoscape 3.9.1 (Shannon et al., 2003) . We identified a small number of candidate genes downstream of *GmCOL1b* that carried strong reliability weights: *LHY/CCA1-LIKE 1* ( *GmLCL1* ) *,* and *LHY/CCA1-LIKE 4* ( *GmLCL4* ), and *TEMPRANILLO 1a* ( *GmTEM1a* ). These candidate genes were predicted to be upregulated by *GmCOL1b* at 25 ^o^ C in LD with respective confidence weights of 0.79, 0.6, and 0.5.

To experimentally verify these predicted regulatory interactions, *GmCOL1b* was expressed in soybean protoplasts and mRNA levels of predicted downstream genes were measured against a non-transfected cell line by RT-qPCR. Protoplasts were isolated and transfected with the *35S:EGFP-GmCOL1b* plasmid DNA (Table 2) as described previously (Wu & Hanzawa, 2018) with minor modifications. Transfected protoplasts were harvested after 24 hours at Zeitgeber time points ZT4-ZT24 with 4-hour intervals. Protoplast viability and GFP expression were examined prior to harvest ( Figure 1B ). Approximately 90% of cells were viable, while about 70% of transfected cells were expressing GFP signals, indicating successful transient expression of GFP-GmCOL1b. *GFP-GmCOL1b* mRNA expression was upregulated by a 50,000-100,000-fold compared to the non-transfected cell line across a 24-hour period. Overexpression of GFP-GmCOL1b upregulated mRNA expression of *GmLCL1, GmLCL4,* and *GmTEM1a* at differing time points *. GmLCL1* exhibited peak expression at ZT8 with a 600-fold increase compared to the negative control, and *GmLCL4* and *GmTEM1a* showed peak expression at ZT12 with a 100-fold and a 400-fold increase compared to the control, respectively.

These results shown above demonstrate that GmCOL1b controls *GmLCL1* , *GmLCL4* , and *GmTEM1a.* Arabidopsis *LHY* and *CCA1* genes encode closely related MYB transcription factors and have a peak expression at dawn (Alabadí et al., 2002; Mizoguchi et al., 2002). In the daytime, *LHY/CCA1* are repressed by *PRR* genes carrying a CONSTANTS, CONSTANS-like and TOC1 (CCT) DNA-binding domain that directly binds to *LHY/CCA1* ’s promoters, creating an essential core oscillator of the circadian clock (Nakamichi et al., 2010; Nakamichi et al., 2012) . Similarly, in soybean, *GmPRR3* genes are shown to directly repress *LHY/CCA1* homologs via their CCT domain (Li et al., 2020; Lu et al., 2020) . CO proteins also contain a CCT domain in their C-terminus (Valverde, 2011; Wu et al., 2014) , thus it is possible that GmCOL1b directly activates *GmLCL1* and *GmLCL4* through its CCT domain. Our observation may be indicative of a feedback regulation between the circadian clock and the flowering gene network via *GmCOL* genes.

Arabidopsis TEMRANILLO 1 (TEM1) and TEM2 belong to the RELATED TO ABI3/VP1 (RAV) subfamily in the AP2 transcription factor family (Castillejo & Pelaz, 2008; Hu et al., 2021) . TEM1 and TEM2 are transcriptional suppressors of *FT* and delay flowering by antagonizing CO, the activator of *FT* (Castillejo & Pelaz, 2008) . In soybean, the TEM homolog’s role in photoperiodic flowering appears to be conserved. GmTEM1b directly interacts with the promoter of the flowering inducer *GmFT5a* and represses its expression, resulting in delayed flowering (Wang et al., 2021) . Our observation may indicate that GmCOL1b may delay flowering through upregulation of *GmTEM* genes.

Our results identified novel target genes of CO in soybean’s photoperiodic flowering and indicated a synergistic function in controlling the circadian clock and flowering transition. Limitations of this study include experimental verification of direct regulation of GmCOL1b on selected candidate genes. However, we cannot exclude indirect regulatory effects on these target genes from unknown factors in the flowering gene network. Nonetheless, this data provides a strategy to further clarify the regulatory roles of CO and the gene regulatory networks controlling the circadian rhythms and flowering transition in crops.

## Methods

**Plant Growth Condition and Sampling**

Williams 82 (PI 518671) seeds were provided by the USDA Soybean Germplasm Collection. Seeds were sown in 6-inch pots containing Sunshine Mix #4 Professional Growing mix with Mycorrhizae and Vermiculite in a 6:1 ratio. The pots were then placed in a growth chamber with 14-hour light exposure per day at 30 °C. The pH and moisture conditions of the soil in each pot were regularly monitored, ensuring the soil moisture range was kept within 40%-50% and the pH at around 7. Fully expanded unifoliate leaves were used for protoplast isolation.

**Plasmid Construction**

The *GmCOL1b* full length CDS ( https://phytozome-next.jgi.doe.gov/report/gene/Gmax_Wm82_a2_v1/Glyma.18G278100 ) was cloned after amplification into the pCR8 vector (Invitrogen), and sequentially sequenced for verification *.* The *GmCOL1b* cDNA was then transferred to the p2FGW7 vector (VIB-UGENT Center for Plant Systems Biology; https://gatewayvectors.vib.be/collection/p2fgw7 by the LR Gateway recombination adhering to the manufacturer’s instructions (Invitrogen). Further information about p2FGW7-35S:EGFP-GmCOL1b plasmid are available upon request by contacting the corresponding author Yoshie Hanzawa (yoshie.hanzawa@csun.edu).

**Protoplast Isolation of *Glycine max***

The protoplast extraction and transfection were conducted according to the procedures described previously with minor modifications (Wu & Hanzawa, 2018) . An enzymatic digestion solution (0.02M of MES pH 5.7, 1.50% w/v Cellulase (R10), 0.50% w/v Macerozyme, 0.20% w/v Pectolyase Y-23, 0.4M D-Mannitol, 0.1M CaCl _2_ , and 7.5% v/v of BSA) was filtered by a 0.45-μ filter. The primary vein and bottom epidermis layer of an 8-day-old soybean unifoliate leaf were removed using the leaf-tape method. The cells were incubated at 22 ^o^ C in low light, with gentle agitation of 100 rpm until digested. The W5 solution (154mM NaCl, 125 mM CaCl _2_ , 5 mM KCl, 2mM MES pH 5.7) was added, and protoplasts were resuspended in MMg solution (4 mM MES pH 5.7, 400 mM D-Mannitol, 15 mM MgCl _2_ ) to a final concentration of 2 x 10 ^5^ mL ^-1^ .

**Protoplast Transfection**

To an aliquot of protoplasts containing 100,000 cells, 20 μg of plasmid DNA was added for transfection, omitting the negative control, mediated by PEG (20% w/v PEG4000, 400 mM D-Mannitol, 100 mM CaCl _2_ ). The cells were resuspended with WI solution (4 mM MES pH 5.7, 500 mM Mannitol, 20mM KCl). To a 6 well tissue culture plate, the wells were coated with 1 mL of 50% v/v sterile calf serum, and protoplasts were placed in the wells. The plate was covered and incubated at 22 ^o^ C overnight in the dark. The protoplasts were visualized with a confocal laser scanning microscope (Leica, USA) to verify transfection efficiency and viability at the time of harvest. Protoplast cells were harvested at ZT4-ZT24 at 4-hour intervals with three biological replications.

**RNA Isolation, cDNA preparation and Real-time Quantitative RT-PCR**

Total RNA was extracted using the Invitrogen RNAqueous Micro-kit for RNA isolation (Invitrogen, CA, USA) following the manufacturer’s instructions. Invasive genomic DNA was removed with the DNAse kit (Invitrogen, CA, USA). First-strand cDNA was synthesized using the iScript cDNA synthesis Kit (BIO-RAD) following the manufacturer’s instructions. The cDNA product was diluted to 1:20 before use. RT-qPCR reaction was performed using QuantStudio3 (Applied Biosystems) with three technical replications. The settings for amplification are a hold at 50 °C for 2 minutes, 95 °C for 10 minutes, a PCR of 95 °C at 15 seconds, ending at 60 °C for 1 minute where amplification was captured with a SYBR target. The results were analyzed on Thermo Fisher’s Connect Data Analysis Apps for real-time qPCR.

## Reagents

**Table:** 

**Accession**	**ID**	**Available From**
Williams 82	PI 518671	USDA

**Table 1.** Soybean accession used for protoplast isolation.

**Table:** 

**Plasmid**	**Gene ID**
p2FGW7-35S:EGFP-GmCOL1b	Glyma.18G278100

**Table 2.** Plasmid name with vector backbone and its respective Gene ID.

**Table:** 

**Primer Name**	**Primer sequence (5’ – 3’)**	**Target Gene Name**
GmPBB2-F GmPBB2-R	TGCCGAAGAAACGCAATGCTTCAA TGCAGCAAGTGAACCTGATCCCAT	*GmPBB2*
GmCOL1b­-F GmCOL1b­-R	CCTAACACCAATAACAATAACA GATCAGTAGTAGCAGCAG	*GmCOL1b*
GmLCL1-F GmLCL1-R	CATGCTTTGAAGAATACGG GTTTTTCTGCATCGCTTCAT	*GmLCL1*
GmTEM1a-F GmTEM1a-R	GCCTACGACATCGCCGCGCA GTCGTAGGTGTGCTTGCGGA	*GmTEM1a*
GmLCL4-F GmLCL4-R	ACATGTTAACCAAGCACTGA AAGAGTAAATACTGCTCCGC	*GmLCL4*

**Table 3.** Primers used in RT-qPCR. Primers were designed targeting exon-exon junctions of targeted genes using the CDS sequences in *Williams82.a2.v1* .

## References

[R1] Alabadí David, Yanovsky Marcelo J., Más Paloma, Harmer Stacey L., Kay Steve A. (2002). Critical Role for CCA1 and LHY in Maintaining Circadian Rhythmicity in Arabidopsis. Current Biology.

[R2] Alcantara Michelle, Acosta Patrick, Azatian Ara , Calderon Carlos , Candray Kevin , Castillo Natalie, Coria-Gomez Luis , Duran Jose , Fam Justina, Hernandez-Segura Diego , Hidalgo Lennix , Huerta Carlos , Jordan Shane , Kagan Kimberly , Loya Karla, Martinez Eduardo, Musaev Kirill , Navarro Roxana, Nazarians Narek , Paglia Robert , Robles Gabriela, Simmons Taylor , Smith Shawn, Soudani Faisel , Valenzuela Emily, Villalobos Jessica , Iftikhar Hira, Hanzawa Yoshie (2022). Experimental Verification of Inferred Regulatory Interactions of EARLY FLOWERING 3 (GmELF3-1) in Glycine max.

[R3] Bu Tiantian, Lu Sijia, Wang Kai, Dong Lidong, Li Shilin, Xie Qiguang, Xu Xiaodong, Cheng Qun, Chen Liyu, Fang Chao, Li Haiyang, Liu Baohui, Weller James L., Kong Fanjiang (2021). A critical role of the soybean evening complex in the control of photoperiod sensitivity and adaptation. Proceedings of the National Academy of Sciences.

[R4] Cao Dong, Li Ying, Lu Sijia, Wang Jialin, Nan Haiyang, Li Xiaoming, Shi Danning, Fang Chao, Zhai Hong, Yuan Xiaohui, Anai Toyoaki, Xia Zhengjun, Liu Baohui, Kong Fanjiang (2015). *GmCOL1a*
and
*GmCOL1b*
Function as Flowering Repressors in Soybean Under Long-Day Conditions. Plant and Cell Physiology.

[R5] Cao Dong, Takeshima Ryoma, Zhao Chen, Liu Baohui, Jun Abe, Kong Fanjiang (2016). Molecular mechanisms of flowering under long days and stem growth habit in soybean. Journal of Experimental Botany.

[R6] Castillejo Cristina, Pelaz Soraya (2008). The Balance between CONSTANS and TEMPRANILLO Activities Determines FT Expression to Trigger Flowering. Current Biology.

[R7] Fornara Fabio, Panigrahi Kishore C.S., Gissot Lionel, Sauerbrunn Nicolas, Rühl Mark, Jarillo José A., Coupland George (2009). Arabidopsis DOF Transcription Factors Act Redundantly to Reduce CONSTANS Expression and Are Essential for a Photoperiodic Flowering Response. Developmental Cell.

[R8] Hayama Ryosuke, Sarid‐Krebs Liron, Richter René, Fernández Virginia, Jang Seonghoe, Coupland George (2017). PSEUDO RESPONSE REGULATORs stabilize CONSTANS protein to promote flowering in response to day length. The EMBO Journal.

[R9] Hu Hongmiao, Tian Shu, Xie Guohui, Liu Rui, Wang Nana, Li Sisi, He Yuehui, Du Jiamu (2021). TEM1 combinatorially binds to
*FLOWERING LOCUS T*
and recruits a Polycomb factor to repress the floral transition in
*Arabidopsis*. Proceedings of the National Academy of Sciences.

[R10] Imaizumi Takato, Schultz Thomas F., Harmon Frank G., Ho Lindsey A., Kay Steve A. (2005). FKF1 F-Box Protein Mediates Cyclic Degradation of a Repressor of
*CONSTANS*
in
*Arabidopsis*. Science.

[R11] Komiya Reina, Ikegami Akiko, Tamaki Shojiro, Yokoi Shuji, Shimamoto Ko (2008). *Hd3a*
and
*RFT1*
are essential for flowering in rice. Development.

[R12] Li Cong, Li Ying-hui, Li Yanfei, Lu Hongfeng, Hong Huilong, Tian Yu, Li Hongyu, Zhao Tao, Zhou Xiaowei, Liu Jun, Zhou Xinan, Jackson Scott A., Liu Bin, Qiu Li-juan (2020). A Domestication-Associated Gene GmPRR3b Regulates the Circadian Clock and Flowering Time in Soybean. Molecular Plant.

[R13] Lin Xiaoya, Liu Baohui, Weller James L., Abe Jun, Kong Fanjiang (2021). Molecular mechanisms for the photoperiodic regulation of flowering in soybean. Journal of Integrative Plant Biology.

[R14] Liu Shulin, Zhang Min, Feng Feng, Tian Zhixi (2020). Toward a “Green Revolution” for Soybean. Molecular Plant.

[R15] Livak Kenneth J., Schmittgen Thomas D. (2001). Analysis of Relative Gene Expression Data Using Real-Time Quantitative PCR and the 2−ΔΔCT Method. Methods.

[R16] Lu Sijia, Dong Lidong, Fang Chao, Liu Shulin, Kong Lingping, Cheng Qun, Chen Liyu, Su Tong, Nan Haiyang, Zhang Dan, Zhang Lei, Wang Zhijuan, Yang Yongqing, Yu Deyue, Liu Xiaolei, Yang Qingyong, Lin Xiaoya, Tang Yang, Zhao Xiaohui, Yang Xinquan, Tian Changen, Xie Qiguang, Li Xia, Yuan Xiaohui, Tian Zhixi, Liu Baohui, Weller James L., Kong Fanjiang (2020). Stepwise selection on homeologous PRR genes controlling flowering and maturity during soybean domestication. Nature Genetics.

[R17] Lu Sijia, Zhao Xiaohui, Hu Yilong, Liu Shulin, Nan Haiyang, Li Xiaoming, Fang Chao, Cao Dong, Shi Xinyi, Kong Lingping, Su Tong, Zhang Fengge, Li Shichen, Wang Zheng, Yuan Xiaohui, Cober Elroy R, Weller James L, Liu Baohui, Hou Xingliang, Tian Zhixi, Kong Fanjiang (2017). Natural variation at the soybean J locus improves adaptation to the tropics and enhances yield. Nature Genetics.

[R18] Mizoguchi Tsuyoshi, Wheatley Kay, Hanzawa Yoshie, Wright Louisa, Mizoguchi Mutsuko, Song Hae-Ryong, Carré Isabelle A., Coupland George (2002). LHY and CCA1 Are Partially Redundant Genes Required to Maintain Circadian Rhythms in Arabidopsis. Developmental Cell.

[R19] Nakamichi Norihito, Kiba Takatoshi, Kamioka Mari, Suzuki Takamasa, Yamashino Takafumi, Higashiyama Tetsuya, Sakakibara Hitoshi, Mizuno Takeshi (2012). Transcriptional repressor PRR5 directly regulates clock-output pathways. Proceedings of the National Academy of Sciences.

[R20] Nakamichi Norihito, Kiba Takatoshi, Henriques Rossana, Mizuno Takeshi, Chua Nam-Hai, Sakakibara Hitoshi (2010). PSEUDO-RESPONSE REGULATORS 9, 7, and 5 Are Transcriptional Repressors in the
*Arabidopsis*
Circadian Clock
&nbsp;. The Plant Cell.

[R21] Sawa Mariko, Nusinow Dmitri A., Kay Steve A., Imaizumi Takato (2007). FKF1 and GIGANTEA Complex Formation Is Required for Day-Length Measurement in
*Arabidopsis*. Science.

[R22] Shannon Paul, Markiel Andrew, Ozier Owen, Baliga Nitin S., Wang Jonathan T., Ramage Daniel, Amin Nada, Schwikowski Benno, Ideker Trey (2003). Cytoscape: A Software Environment for Integrated Models of Biomolecular Interaction Networks. Genome Research.

[R23] Song Young Hun, Kubota Akane, Kwon Michael S., Covington Michael F., Lee Nayoung, Taagen Ella R., Laboy Cintrón Dianne, Hwang Dae Yeon, Akiyama Reiko, Hodge Sarah K., Huang He, Nguyen Nhu H., Nusinow Dmitri A., Millar Andrew J., Shimizu Kentaro K., Imaizumi Takato (2018). Molecular basis of flowering under natural long-day conditions in Arabidopsis. Nature Plants.

[R24] Song Young Hun, Shim Jae Sung, Kinmonth-Schultz Hannah A, Imaizumi Takato (2015). Photoperiodic Flowering: Time Measurement Mechanisms in Leaves. Annual Review of Plant Biology.

[R25] Song Young Hun, Smith Robert W., To Benjamin J., Millar Andrew J., Imaizumi Takato (2012). FKF1 Conveys Timing Information for CONSTANS Stabilization in Photoperiodic Flowering. Science.

[R26] Takagi Hiroshi, Hempton Andrew K., Imaizumi Takato (2023). Photoperiodic flowering in Arabidopsis: Multilayered regulatory mechanisms of CONSTANS and the florigen FLOWERING LOCUS T. Plant Communications.

[R27] Turck Franziska, Fornara Fabio, Coupland George (2008). Regulation and Identity of Florigen: FLOWERING LOCUS T Moves Center Stage. Annual Review of Plant Biology.

[R28] Valverde F. (2011). CONSTANS and the evolutionary origin of photoperiodic timing of flowering. Journal of Experimental Botany.

[R29] Valverde Federico, Mouradov Aidyn, Soppe Wim, Ravenscroft Dean, Samach Alon, Coupland George (2004). Photoreceptor Regulation of CONSTANS Protein in Photoperiodic Flowering. Science.

[R30] Wang Yuhe, Xu Chongjing, Sun Jiafan, Dong Lidong, Li Minmin, Liu Ying, Wang Jianhui, Zhang Xiaoming, Li Dongmei, Sun Jingzhe, Zhang Yuntong, Shan Jinming, Li Wenbin, Zhao Lin (2021). *GmRAV*
confers ecological adaptation through photoperiod control of flowering time and maturity in soybean. Plant Physiology.

[R31] Wu Faqiang, Hanzawa Yoshie (2018). A Simple Method for Isolation of Soybean Protoplasts and Application to Transient Gene Expression Analyses. Journal of Visualized Experiments.

[R32] Wu Faqiang, Kang Xiaohan, Wang Minglei, Haider Waseem, Price William B., Hajek Bruce, Hanzawa Yoshie (2019). Transcriptome-Enabled Network Inference Revealed the GmCOL1 Feed-Forward Loop and Its Roles in Photoperiodic Flowering of Soybean. Frontiers in Plant Science.

[R33] Wu Faqiang, Price Brian William, Haider Waseem, Seufferheld Gabriela, Nelson Randall, Hanzawa Yoshie (2014). Functional and Evolutionary Characterization of the CONSTANS Gene Family in Short-Day Photoperiodic Flowering in Soybean. PLoS ONE.

[R34] Xia Zhengjun, Watanabe Satoshi, Yamada Tetsuya, Tsubokura Yasutaka, Nakashima Hiroko, Zhai Hong, Anai Toyoaki, Sato Shusei, Yamazaki Toshimasa, Lü Shixiang, Wu Hongyan, Tabata Satoshi, Harada Kyuya (2012). Positional cloning and characterization reveal the molecular basis for soybean maturity locus
*E1*
that regulates photoperiodic flowering. Proceedings of the National Academy of Sciences.

[R35] Yano Masahiro, Katayose Yuichi, Ashikari Motoyuki, Yamanouchi Utako, Monna Lisa, Fuse Takuichi, Baba Tomoya, Yamamoto Kimiko, Umehara Yosuke, Nagamura Yoshiaki, Sasaki Takuji (2000). *Hd1*
, a Major Photoperiod Sensitivity Quantitative Trait Locus in Rice, Is Closely Related to the Arabidopsis Flowering Time Gene
*CONSTANS*. The Plant Cell.

[R36] Yu Jae-Woong, Rubio Vicente, Lee Na-Yeoun, Bai Sulan, Lee Sun-Young, Kim Sang-Sook, Liu Lijing, Zhang Yiyue, Irigoyen María Luisa, Sullivan James A., Zhang Yu, Lee Ilha, Xie Qi, Paek Nam-Chon, Deng Xing Wang (2008). COP1 and ELF3 Control Circadian Function and Photoperiodic Flowering by Regulating GI Stability. Molecular Cell.

